# Oral health needs and use of services by adults: evidence from SB Brasil 2023

**DOI:** 10.1590/1807-3107bor-2025.vol39.0049

**Published:** 2025-05-19

**Authors:** Rafaela da Silveira PINTO, Andreia Maria Araújo DRUMMOND, Raquel Conceição FERREIRA, Viviane Elisângela GOMES, Alcir José de OLIVEIRA, Amanda Pinto Bandeira de Sousa MARQUES, Anna Rachel dos Santos SOARES, Mariane Carolina Faria BARBOSA, Rosa Núbia Vieira de MOURA

**Affiliations:** (a) Universidade Federal de Minas Gerais – UFMG, School of Dentistry, Department of Social and Preventive Dentistry, Belo Horizonte, MG, Brazil.; (b) Ministério da Saúde, General Coordination of Oral Health, Brasília, DF, Brazil.; (c) Universidade Federal de Minas Gerais – UFMG, School of Dentistry, Department of Child and Adolescent Oral Health, Belo Horizonte, MG, Brazil.

**Keywords:** Adults, Oral Health, Dental Assistance, Self-perception, Oral Health Surveys

## Abstract

This cross-sectional study investigated the demand for and use of dental services, use of prosthesis, self-perception and clinical evaluation of the need for dental treatment and prostheses in adults aged 35 to 44 years participating in SB Brasil 2023. Demographic and contextual variables were analyzed, as well as clinical variables and self-perception of oral health (treatment and prosthesis). Statistical analysis was conducted using SPSS Statistics 23.0 software. The results showed that the majority of adults used dental services in the past year, mainly in the private and public sectors. Among public service users, 62.3% were actually attended to. The need for prostheses was identified in 49.6% of individuals who do not use prostheses and in 77.7% of those who already use them. It was observed that 86.6% of individuals who self-assessed their need for prosthesis were also clinically evaluated as needing one. Regarding dental treatment, this evaluation coincided in 65.6% of individuals. Disparities in access to and utilization of dental services were identified, as well as a high demand for prosthetic care and rehabilitation. These results emphasize the need for public policies that expand access to and improve the quality of dental services in adult healthcare in Brazil. Furthermore, they suggest that promoting self-perception could be an effective strategy to improve planning and adherence to oral health care.

## Introduction

Oral disorders, such as dental caries, periodontitis, and tooth loss, affect approximately 44.5% of the global population.^1^ These conditions cause serious functional and social disabilities, such as difficulty chewing, pain, severe infections, harm to interpersonal relationships, and reduced quality of life. Additionally, they have a significant economic impact, exacerbating social inequalities due to the high cost of dental treatments and loss of productivity at work.^2-5^

These disorders are chronic and progressive, tending to worsen with aging.^1^ In Brazil, although oral health in children and young people has improved in recent decades, the situation for adults remains concerning.^6,7^ National surveys show that this age group experiences high tooth loss and an increasing need for prosthetic rehabilitation.^7,8^ In 2010, Brazilian adults had an average DMFT (Decayed, Missing, and Filled Teeth) index of 20.4 teeth, with 75.2% requiring dental treatment, and only 31.2% did not need dental prostheses, highlighting the high demand for dental care.^7^

Despite this demand, access to dental services for adults remains limited. The main challenges include the high cost of treatments, inaccessible hours, and financial restrictions in public services, which often prioritize medical care over dental care.^1,9^ Since the launch of the National Oral Health Policy in 2004, the Brazilian Ministry of Health has been working to expand access to treatment, decentralizing services, and expanding specialized care through the implementation of Specialized Dental Centers (Centros de Especialidades Odontológicas - CEO), Regional Dental Prosthetics Laboratories, and water fluoridation in public water supplies.^10-13^

To evaluate the effectiveness of these public policies, it is essential to understand the current state of oral health in Brazilian adults. The national oral health survey, SB BRASIL 2023, provided updated clinical data on treatment and prosthetic needs, as well as investigating participants’ self-perception of oral health ^14^. Self-perception is a simple, cost-effective measure that complements clinical assessments.^15^ In this context, this study describes the demand for and use of dental services, prosthesis use, self-perception, and clinical evaluation of the need for dental treatment and prostheses in Brazilian adults.

## Methods

### Study design and population

This is a cross-sectional observational study, conducted with secondary data from the National Oral Health Survey - SB Brasil 2023.^14^The main methodological aspects of the study are available in the Technical Report, accessible online.^14,16^ The sample, representative at the national level, consists of individuals aged 35 to 44, totaling 9019 participants. This age group is considered the standard for evaluating oral health conditions in adults.^14^

The SB Brasil 2023 Project was approved by the National Research Ethics Committee (Opinion No. 4.823.054), following the ethical principles of Resolution 466/12 of the National Health Council. Participation in the study was voluntary, with the signing of the Informed Consent Form (ICF).

### Variables

Sociodemographic variables, as well as variables of access to and use of oral health services, along with self-perception of oral health, were obtained through a standardized questionnaire applied to the participants. The analyzed variables included: sex (male; female), color/race (white; non-white; doesn’t know/did not answer), education (Did not attend school/adult education; Complete/incomplete basic education; Complete/incomplete high school; Complete/incomplete higher education; Doesn’t know/did not answer), participation in social programs (No; Yes; Doesn’t know/Did not answer), and access to water at home (Piped in at least one room; Piped only on the land/property; Not piped; Doesn’t know/Did not answer).

Self-perception of the need for treatment (No; Yes; Doesn’t know/Did not answer) and self-perception of the need for prostheses (No; Yes; Doesn’t know/Did not answer) were also assessed. In addition, the need for treatment was recorded based on the evaluation of the dentist, performed according to the index recommended by the WHO.^17^ This last variable was dichotomized as the need for treatment (No/Yes).

The evaluation of the use and need for prostheses was conducted through clinical assessment by the dentist, following the WHO guidelines ^17^. In the clinical evaluation of the need for prostheses, quality was considered and assessed by the Prosthesis Quality Index.^18^ Information on the use and need for prostheses was dichotomized (No/Yes).

Regarding the search for and use of dental services, the investigation included: search for service (Did not seek; Sought and was not attended to; Sought and was rescheduled to another day/location; Sought and was attended to; Doesn’t know/Did not answer), type of service sought (Did not seek; Public service; Private service; Health insurance or private healthcare plan; Other; Doesn’t know/Did not answer), frequency of service use (Has never been to the dentist; Up to 1 year; More than 1 up to 2 years; More than 2 up to 3 years; More than 3 years; Doesn’t know/Did not answer), and type of service used (Has never been to the dentist; Public service; Private service; Health insurance or private healthcare plan; Other; Doesn’t know/Did not answer).^16^

### Statistical analysis

Descriptive analyses were performed and presented in the form of percentages and 95% confidence intervals (CI95%). The data were analyzed using SPSS Statistics 23.0 (IBM Corp., Armonk, USA) with the Complex Samples module.

## Results

This study was conducted with 9,019 adults who participated in the SB Brasil 2023 survey. The majority were female, self-reported as non-white, and had complete or incomplete High School level. Regarding dental service utilization in the past year, 42.5% of adults sought care and were successfully attended to, while 10.9% sought care but were not attended to. In terms of service usage, 49.6% of participants reported having used some dental service in the past year, with the private sector (44.7%) and public sector (40.8%) being the most commonly sought.

In terms of self-perception, 72.3% considered themselves in need of dental treatment, and 53.6% believed they needed dental prostheses. In the clinical evaluation, 55.2% were diagnosed with the need for treatment, while 53.6% required prosthetic rehabilitation. Details of these descriptive analyses are presented in Table 1.

**Table 1:** Description of adults according to sociodemographic characteristics, use of service, oral health self-perception, and need for treatment.

Variables	n	% (95CI%)
Sex		
Male	2,876	33.7 (31.2–36.3)
Female	6,143	66.3 (63.7–68.8)
Race		
White	2,848	41.7 (37.7–45.7)
Non-white	6,093	57.0 (53.0–60.9)
Doesn’t know/Did not answer	78	1.3 (0.7–2.4)
Level of education		
Did not study/Adult education	216	2.9 (1.8–4.6)
Basic Education incomplete/complete	1,762	21.0 (18.2–24.0)
High School incomplete/complete	4,389	49.6 (45.6–53.6)
Higher Education incomplete/complete	2,551	24.6 (21.1–28.6)
Doesn’t know/Did not answer	101	1.9 (0.9–4.0)
Receives financial resources from social programs		
No	5,146	62.3 (58.3–66.1)
Yes	3,301	30.8 (27.5–34.2)
Doesn’t know/Did not answer	572	6.9 (4.6–10.3)
Water		
Piped in at least one room	7,399	83.6 (19.6–87.0)
Piped only on the land or property	1,023	11.3 (8.4–14.9)
Not piped	250	1.9 (1.1–3.4)
Doesn’t know/Did not answer	347	3.2 (2.1–4.8)
Sought service in the last year		
Did not seek	3,478	36.0 (32.9–39.1)
Sought service but was not attended to	849	10.9 (8.8–13.5)
Sought service and was scheduled for another day/location	700	8.6 (6.5–11.4)
Sought service and was attended to	3,894	42.5 (39.2–45.9)
Doesn’t know/Did not answer	98	2.0 (0.9–4.2)
Type of service sought in the last year		
Did not seek	3,478	36.0 (32.9–39.1)
Public care	2,730	29.1 (25.8–32.7)
Private care	2,209	26.9 (23.6–30.5)
Health insurance or private healthcare plan	454	5.6 (4.1–7.5)
Other	30	0.3 (0.1–0.7)
Doesn’t know/Did not answer	118	2.2 (1.0–4.4)
Use of service		
Has never been to the dentist	101	0.9 (0.4–1.7)
Up to 1 year	4,447	49.6 (46.1–53.0)
More than 1 year, up to 2 years	1,781	19.0 (17.0–21.2)
More than 2 years, up to 3 years	1,030	12.1 (10.0–14.4)
More than 3 years	1,331	14.5 (12.4–17.0)
Doesn’t know/Did not answer	329	4.0 (2.5–6.2)
Type of service used		
Has never been to the dentist	101	0.9 (0.4–1.7)
Public care	4,131	40.8 (35.8–46.1)
Private care	3,674	44.7 (40.3–49.3)
Health insurance or private healthcare plan	661	8.2 (6.2–10.7)
Other	56	0.6 (0.3–1.5)
Doesn’t know/Did not answer	396	4.8 (3.2–7.0)
Self-perception of the need for treatment		
No	2,069	23.6 (10.5–27.0)
Yes	6,604	72.3 (68.1–76.2)
Doesn’t know/Did not answer	346	4.1 (2.6–6.3)
Self-perception of the need for prosthesis		
No	7471	82.9 (80.3–85.3)
Yes	1401	15.5 (13.3–17.9)
Doesn’t know/Did not answer	147	1.6 (1.0–2.5)
Need for treatment*		
No	3,934	44.8 (41.1–48.5)
Yes	5,053	55.2 (51.5–58.9)
Need for prosthesis*		
No	4,001	46.4 (42.1–50.9)
Yes	4,983	53.6 (49.1–57.9)
Use of prosthesis*		
No	7,749	85.9 (83.7–87.8)
Yes	1,220	14.1 (12.2–16.3)

*Patients who were not clinically assessed or with no response were not counted in the percentage presented.

It was observed that self-perception and clinical evaluation for the need for prostheses coincided in 86.6%. For the need for dental treatment, this percentage was 65.6%. Additionally, 76.0% of individuals who considered themselves not in need of treatment were clinically assessed as not needing it.

A high demand for dental prosthetic services among adults was noted. Of the participants not using prostheses, 49.6% were diagnosed with a current need, and among those already using prostheses, 77.7% still required some form of prosthetic intervention (Table 2).

**Table 2:** Sample distribution and percentages of use *versus* need for prosthesis; of the self-perception of the need for prosthesis *versus* the need for prosthesis assessed by examiners; and of the self-perception of the need for treatment *versus* the need for treatment assessed by examiners.

Variables	Need for prosthesis			
	No		Yes	
	n	% (95%CI)	n	% (95%CI)
Use of prosthesis				
No	3,734	50.4 (45.4–55.4)	4,015	49.6 (44.6–54.6)
Yes	256	22.3 (15.9–30.3)	964	77.7 (69.7–84.1)
Self-perception of the need for prosthesis				
No	3,854	52.9 (48.0–57.7)	3,607	47.1 (42.3–52.0)
Yes	105	13.4 (8.0–21.5)	1,294	86.6 (78.5–92.0)
Self-perception of the need for treatment				
No	1,540	76.0 (70.8–80.4)	525	24.0 (19.6–29.2)
Yes	2,216	34.4 (29.9–39.3)	4,384	65.6 (60.7–70.1)

Responses with the option ‘doesn’t know/did not answer’ were not included in the table.

Among those who sought public services in Brazil, 62.3% received care, 19.5% did not receive care, and 18.2% were scheduled for another time. In the private sector, 73.3% received immediate care, 26.9% were not attended to, and 9.8% were scheduled for another time (Table 3).

**Table 3:** Distribution of the types of dental services sought and adult treatment outcomes in the last year.

Type of service sought	Have they sought any dental service in the last year?							
	Did not seek		Sought but was not attended to		Sought but was scheduled for another day/location		Sought and was attended to	
	n	% (95%CI)	n	% (95%CI)	n	% (95%CI)	n	% (95%CI)
Did not seek	3,478	100 (100.0-100.0)	-	-	-	-	-	-
Public service	-	-	567	19.5 (15.4-24.3)	367	18.2 (12.9-24.3)	1,796	62.3 (56.3-67.9)
Private service	-	-	244	26.9 (12.5-22.6)	254	9.8 (6.9-13.7)	1,711	73.3 (67.6-78.3)
Health insurance or plan	-	-	29	11.9 (3.5-33.1)	71	10.6 (6.3-17.5)	353	77.5 (63.5-87.2)
Other	-	-	5	7.3 (1.8-25.1)	7	32.9 (7.1-76.0)	18	59.8 (21.2-89.1)

Responses with the option ‘doesn’t know/did not answer’ were not included in the table.

Table 4 shows the distribution of the locations of the last dental consultation and the time elapsed since then. The data indicate differences in access and frequency of consultations depending on the type of service used. Among public service users, 52.9% had a consultation in the last 12 months. Among private service users, this percentage was 50.3%, and for health insurance and plans, it was 64%.

**Table 4:** Distribution of the types of services where the last dental visit was conducted and the time elapsed.

Type of service where the last dental visit was conducted	When was the last dental visit?									
	Has never been to the dentist		Up to 1 year		> 1, up to 2 years		> 2, up to 3 years		More than 3 years	
	n	% (95%CI)	n	% (95%CI)	n	% (95%CI)	n	% (95%CI)	n	% (95%CI)
Has never been to the dentist	101	100 (100.0–100.0)	–	–	–	–	–	–	–	–
Public service	-	–	2,007	52.9 (48.1–57.7)	839	19.5 (16.5–22.9)	550	12.3 (9.7–15.5)	734	15.2 (12.1–19.0)
Private service	-	–	1,985	50.3 (45.9–54.7)	803	21.2 (18.4–24.3)	393	12.7 (9.2–17.2)	492	15.8 (12.3–20.1)
Health insurance or plan	-	–	423	64.0 (50.8–75.4)	115	13.0 (8.7–19.0)	66	14.8 (7.3–27.6)	57	8.2 (3.7–17.1)
Other	-	–	24	30.3 (8.4–67.2)	6	52.0 (16.2–85.8)	6	3.3 (0.8–12.6)	20	14.5 (4.4–38.3)

Responses with the option ‘doesn’t know/did not answer’ were not included in the table.

## Discussion

Recently, the National Oral Health Policy, known as *Brasil Sorridente*, was included in the Brazilian Organic Health Law, becoming an integral part of the Unified Health System (SUS). This inclusion reaffirmed the obligation and guarantee of universal, equitable, and continuous access to oral health services for the entire Brazilian population.^19,20^ Furthermore, the Oral Health Care Network was established and included in the National Oral Health Policy.^10^ Despite these advances, recent studies indicate that the adult population still faces significant barriers to accessing dental services and has a high demand for treatments.^9,12,14^In this context, this study is innovative in providing updated data on oral health, collected in the national epidemiological survey of 2023. The results highlight that, although there has been progress in the coverage of oral health teams, the annual frequency of service utilization remains below 50% among adults. Additionally, there is a high prevalence of the need for dental treatment and prosthetic rehabilitation in this age group.^14^

When comparing the results of SB Brasil 2023 with those of SB Brasil 2010, a significant improvement in the oral health of the adult population is observed. The average DMFT index reduced from 16.75 to 10.7 affected teeth.^7,14^ In 2023, the average number of missing teeth, a component of the DMFT index, was 3.45 teeth.^14^ Tooth loss, in addition to being a major public health issue due to the functional and social consequences it causes, also represents a challenge for public health planning, especially when it occurs prematurely in adults and young people^4^. Historically, dental practice in Brazil has been marked by a curative model based on surgical-restorative treatments. This model contributed to high rates of edentulism, which are still observed as a legacy of this model of care.^21,22^

The assessment of the need for and use of dental prostheses is essential for understanding the impact of tooth loss and edentulism, as well as for estimating the severity of the problem, providing support for the planning of public policies based on the real needs of the population.^16^ In this study, more than 50% of the adults assessed required dental prosthesis. Although these figures are still high, they show a reduction compared to 2010, when 68.8% needed prostheses.^7,14^ This improvement is believed to be primarily due to the reduction in the DMFT index in this age group, as the demand for prosthetic rehabilitation remains high even among individuals already using prostheses (77.7%), despite the increased availability of secondary services. These results reinforce the need to expand the provision of prosthetic rehabilitation services to adequately meet the population’s demand.

Thus, policies aimed at expanding specialized services and Regional Dental Prosthesis Laboratories (LRPDs) should be encouraged, considering that political, operational, administrative, financial factors, and the interest of social control directly influence the allocation and functioning of LRPDs. Additionally, the utilization of dental services does not depend solely on their availability or clinical necessity, since individuals’ self-perception on their health and the perceived need for treatment plays a crucial role in the demand for services.^23^

A study on the implementation of the *Brasil Sorridente* program between 2001 and 2015 highlighted a more than 470% growth in the number of oral health teams in the Family Health Strategy, a 4% increase in primary care, while specialized services saw increases of over 200%. During this period, population coverage rose from 9% to 43% in Brazil.^13^. By September 2023, Brazil had 29,618 oral health teams, covering approximately 91 million people.^14^ Although Brazil has made significant advances in oral health coverage, it is estimated that approximately 120 million Brazilians remain without access to public dental care. In this context, the Previne Brasil program has one of its goals to reduce gaps in care and waiting times through expanded primary care. To this end, the Ministry of Health plans to implement 3,030 new oral health teams by 2026.^24^

In this study, about 55% of adults reported need for dental treatment, reflecting the high demand for care in this age group. The provision of health services in Brazil is marked by regional and social inequalities, making addressing this scenario a complex challenge. Evaluating access to dental services requires identifying barriers related to their availability, considering a set of factors that include causal pathways, social inequalities, and other determinants that impact the oral health of the population.^25-27^

Moreover, only about 45% of participants reported using dental services in the past year. This result is similar to what was observed in the 2013 National Health Policy, where 44.4% of Brazilians used dental services in the last twelve months, with significant disparities, marked by lower use among Black individuals and those without formal education.^25,28^ Despite the expansion of dental services in the past decade, these data indicate a stagnation in the access and utilization of services in Brazil. This scenario may reflect a history where dentistry was neglected by public health policies in Brazil, with a care model focused on emergency treatments and priority groups ^25,29^.

The importance of regular consultations for maintaining oral health is well described in the literature, highlighting that the absence of frequent and routine appointments can hinder the early identification of oral health problems and the continuity of necessary treatments.^30,31^ Therefore, it is essential to develop educational strategies aimed at informing the population about the importance of continuous dental treatments, as well as the chronic and often asymptomatic nature of some oral conditions. These actions can prevent the worsening of oral problems, which, if neglected, may result in complications in the future.^15^

Among users who sought care from public services, only 62.3% were actually attended to. The results indicate that, although the *Brasil Sorridente* program has expanded the national coverage of dental services, challenges in improving retention and continuity of treatment still exist.^25,32^ This perpetuates disparities in access and utilization of dental services, as well as a significant demand for continuous care and prosthetic rehabilitation.

In this study, considerable accuracy between self-perception and clinical evaluation of the oral health of the individuals examined was highlighted. Health self-perception is a concept that involves the individual’s understanding of their health status, considering the sociocultural elements of their context. Collected through questionnaires or interviews, self-perception is recognized as a practical and economical method, especially for large-scale epidemiological surveys like SB Brasil. Due to its simplicity, it can be used as a complementary indicator to the information obtained from clinical health exams.^22,33-35^

A study using data from SB Brasil 2010 identified a 78.8% concordance between self-perception and clinical evaluation in adults in Brazil.^15^ In this study, 77% and 86% of individuals who were self-assessed as needing prosthetics and dental treatment evidenced their need during the clinical evaluation. Costa et al. (2013) found a 76.9% concordance between satisfactory quality according to the professional assessment and user satisfaction with complete prostheses. However, when prostheses were assessed as unsatisfactory, the concordance was considerably lower, reaching only 39%.^36^ These findings emphasize the importance of considering both the clinical perspective and the user’s perception in assessing oral health. It is worth noting that the results suggest that the dentist overestimated the need for prosthetic rehabilitation, and the patient overestimated the need for dental treatment. These factors may impact on the demand for services, increasing costs and affecting their availability for patients who truly need them.

Among the limitations of this study is its cross-sectional design, which prevents establishing causal relationships between the factors analyzed. Despite this, it is important to note that this is a nationally representative study, which adds robustness to the results and, by using similar parameters, allows comparison with the previous survey, SB Brasil 2010. Another limitation relates to the use of secondary data. The results presented in this study are important for improving public oral health policies, contributing to the planning, organization, and monitoring of services. Moreover, they provide insights for formulating strategies that expand access and promote equity in national dental care.

## Conclusion

Disparities in access to and utilization of dental services were identified, along with a significant demand for care and prosthetic rehabilitation among Brazilian adults. These results reinforce the need for public policies aimed at expanding access to and improving the quality of dental services in adult healthcare in Brazil. Furthermore, they suggest that valuing self-perception could be an effective strategy to enhance planning and adherence to oral health care.
